# Supplementary material for: A generative model of the connectome with dynamic axon growth
Source: Netw Neurosci. 2024 Dec 10;8(4):1192–211. doi: 10.1162/netn_a_00397 (PMC11674315; doi:10.1162/netn_a_00397)
Supplement: Supplementary file 1 [file netn-8-4-1192-s001.pdf]

Supplementary Materials

A generative model of the connectome with dynamic axon growth

Yuanzhe Liu [a, b], Caio Seguin [b, c], Richard F. Betzel [c],
Daniel Han [d], Danyal Akarca [e, f], Maria A. Di Biase [b, g], Andrew Zalesky [a, b]

a. Department of Biomedical Engineering, Faculty of Engineering & Information Technology, The University of Melbourne, Melbourne, VIC, Australia
b. Melbourne Neuropsychiatry Centre, Department of Psychiatry, The University of Melbourne, Melbourne, VIC, Australia
c. Department of Psychological and Brain Sciences, Indiana University, Bloomington, IN, USA
d. School of Mathematics and Statistics, University of New South Wales, Sydney, NSW, Australia
e. MRC Cognition and Brain Sciences Unit, University of Cambridge, UK
f. Department of Electrical and Electronic Engineering, Imperial College London, UK
g. Department of Psychiatry, Brigham and Women's Hospital, Harvard Medical School, Boston, MA, USA

Corresponding authors:

Yuanzhe Liu (yuanzhel@student.unimelb.edu.au)

Andrew Zalesky (azalesky@unimelb.edu.au)

Node center definition

When defining the nodes, we first sample node centers, after which node regions are assigned through a 1D Voronoi Tessellation with sampled node centers being the seeds. The defined node centers can be viewed as effective centers of attractive forces, which guarantees the greatest attractive force that any given point on the perimeter receives is always delivered by the node it belongs to, and points located at the boundary of two regions receive equal forces from the two nodes. However, Voronoi cells are not necessarily symmetric on both sides of the seeds; in other words, sampled node centers are not the geometric centers of parcellated brain regions.

To investigate this issue, we tested the impact of using geometric centers in our model. Specifically, with the representative parameter combinations of $\beta = 1$ and $L_s = 1$, we generated networks using the effective centers reported in the main (denoted by C) and the geometric centers (denoted by C_g) and compared the results. As shown in Fig. S1, the axon architecture generated by corresponding C and C_g pairs are visually alike (Fig. S1a, b), and both generated networks display a negatively correlated weight and distance, lognormal weight distributions, and scale-free degree distributions. These results suggest that using the geometric center of parcellations for axon simulation also generates brain-like networks. Options for simulating networks with both C and C_g are available in the released MATLAB code.

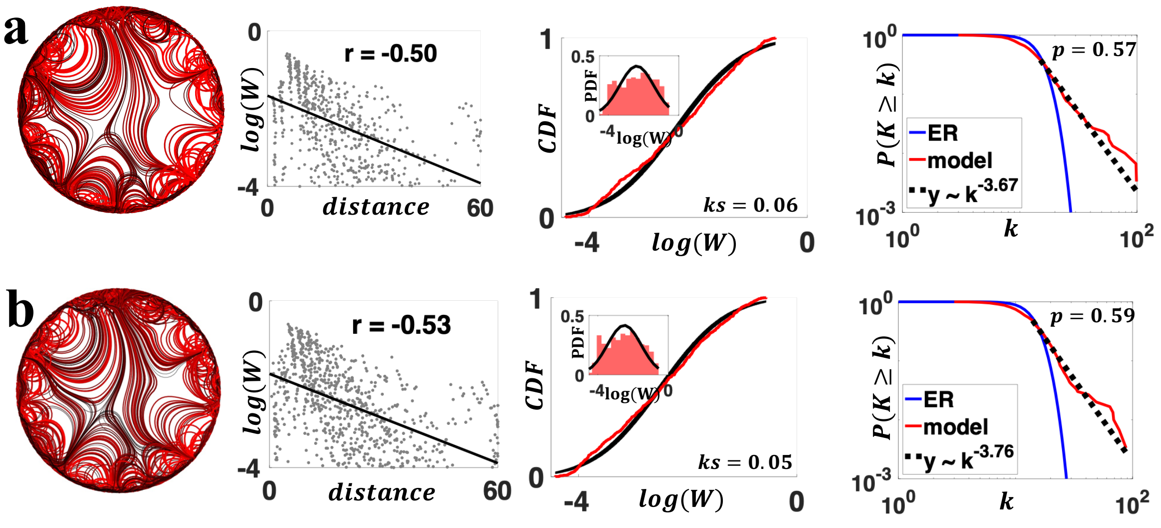

Figure S1. Compare networks generated with C_g and C. a) The axon architecture (left), weight-distance association (center-left), and weight distribution (center-right) of an example network generated with C_g. The right plot describes the scale-free degree distribution of networks generated with C_g (the network with the median scale-free score among 1000 generations). b) Same as a) but for networks generated with C. Note that the example network was kept with the same node segment parcellation as in a), and the generated axon architectures are visually alike.

Constraints on axon growth directions

The model specified the maximum angular disparity θ an axon can make at each increment step. Fig. S2 illustrates how this angular constraint is applied.

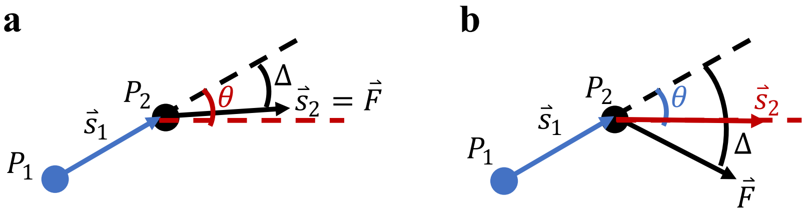

Figure S2. Angular constraint θ. *a) axons grow in the direction of the summed force if the computed angular difference is smaller than or equal to θ. In growth step $i-1$, the axon grew from point P1 (blue dot) to point P2 (black dot), and its growth course in step $i-1$ is $\vec{S}_1$ (blue arrow). The black arrow denoted the summed force $\vec{F}$ that the axon received at position P2. Δ*

represented the angular difference between $\vec{F}$ and $\vec{S}_1$, and the angle θ between black and red dashed lines was the maximum angular disparity allowed. When $\Delta \leq \theta$, the axon's growth direction in step i ($\vec{S}_2$) equals $\vec{F}$. b) Same as a) but for $\Delta > \theta$, the angular difference between $\vec{S}_1$ and $\vec{S}_2$ (red arrow) is forced to θ.

In addition, two special cases were considered.

Case 1: if $\vec{\mathrm{F}}$ and $\vec{s}_1$ are in opposite direction ($\frac{\vec{\mathrm{F}}}{|\vec{F}|} + \frac{\vec{s}_1}{|\vec{s}_1|} = 0$), the clockwise/counterclockwise direction of θ could not be determined. In this scenario, we forced $\vec{s}_2$ to be in the direction of $\vec{s}_1$.

Case 2: if $\vec{F}$ has a magnitude of 0, its direction cannot be determined. In this scenario, the model randomly sampled a non-zero $\vec{F}$, after which the angular constraint θ was applied.

"Black hole" region and β values

In Results and Methods, we argued that growing axons could fail to land on the circle circumference because they were trapped within a "black hole" region. This happened when the values of β were small, such that the guidance from local nodes was too weak to allow axon terminations. As shown in Fig. S3, as β increases, trapped axons suddenly escape to form middle-range to long-range connections. However, if β is too large, long-range connections cannot form because of the strong attractive force from local nodes. This explains why small variations in β leads to dramatic change in generated networks: only a small critical range of β allows long-range connections to form.

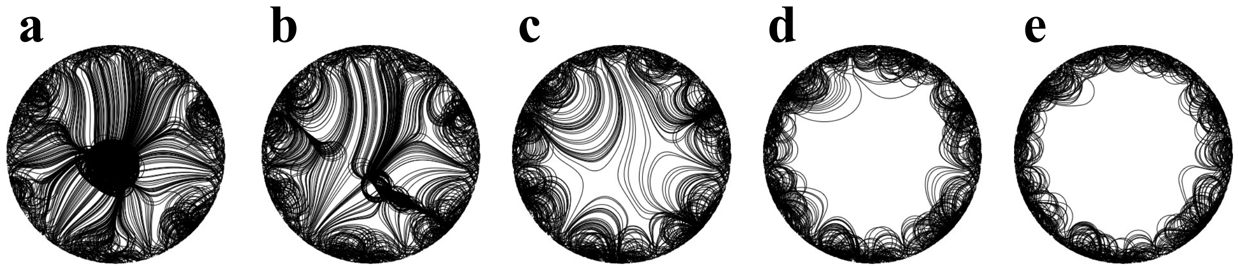

Figure S3. Effect of β on axon length. *a)-e) each displayed a subset of 1,000 simulated axons from networks shown in Fig. 2a. $\beta = 0.98, 0.99, 1, 1.01$, and, 1.02 respectively (from a to e). a) A "black hole" region is evident with small values of β. Axons were trapped and failed to form long-range connections. b)-c) As β increases, long-range connections start to appear. d)-e) If β is too large, long-range connections disappear due to strong local attractive forces.*

The effect of excluded axons

When constructing networks we exclude unsuccessful axons and self-connections. In the results, we observed that larger L_s corresponds to denser networks, and this can potentially be the result of removing more within-node connections when L_s is small. With this concern, we evaluate and visualize the proportion of simulated axons leading to i) self-connections, ii)_unsuccessful connections, and iii) successful connections, i.e., axons that are kept for network construction. The results are shown in Fig. S4. Though excluded self-connections and unsuccessful axons can be an issue when $\beta = 0.98$ (54% of axons are unsuccessful) and $L_s = 0.1$ (91% of axons are self-connections), the number of removed connections is generally small (less than 2% of simulated axons) for other evaluated parameter combinations. These results suggest that the number of excluded axons is not the major driver of variations in network density, especially near the parameter combinations that generate connectome-like networks. It is the increasing L_s that increases the network density by promoting the formation of new connections that are absent in smaller L_s.

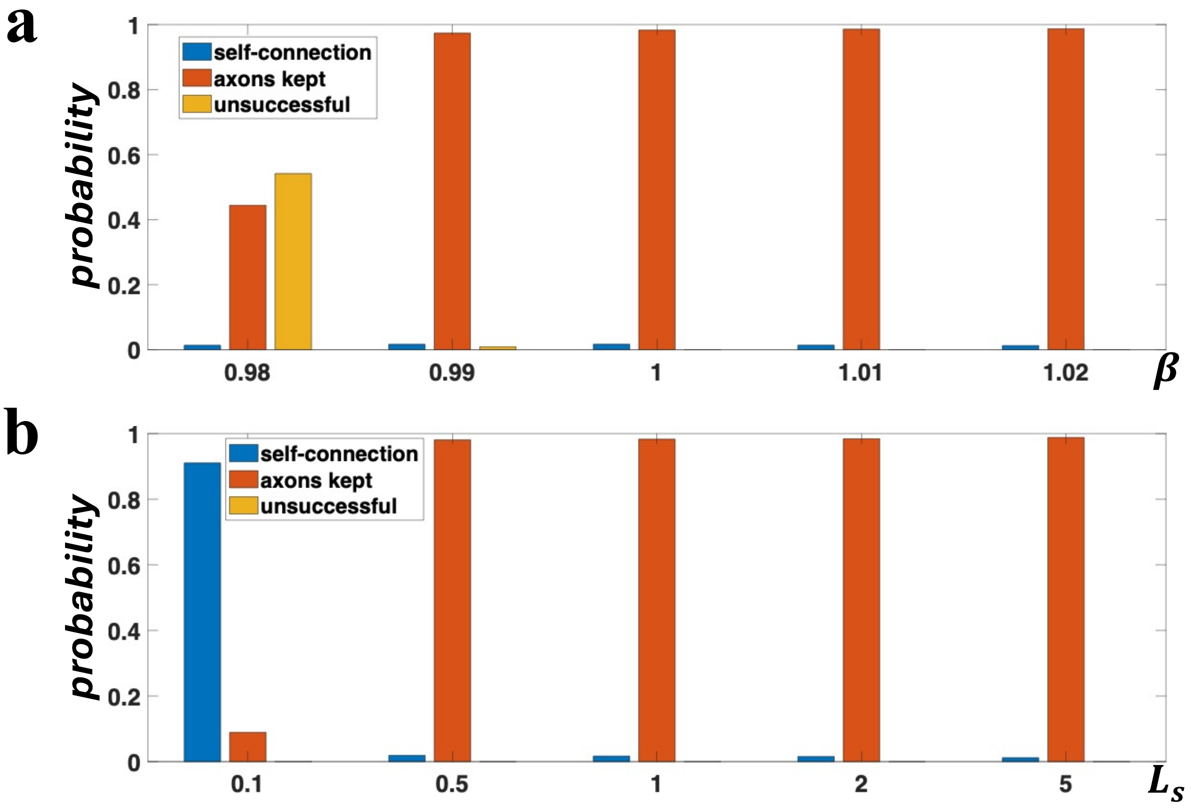

Figure S4. Excluded axons are not the major driver of variations in network density. a) The probability of an axon being removed (self-connection and unsuccessful axons) in networks generated with a variety of β. b) The probability of axon being removed in networks generated with a variety of L_s. Variations in network density are evident between $L_s = 0.5, 1, 2, 5$ (11, 24, 35, 75% network densities, respectively), despite the number of removed axons being small (<2%) and comparable.

Parameter specification

Eight model parameters were defined in the model. In this study, we were interested in variations in β and L_s; other parameters were fixed. Here we provided a brief justification to this implementation.

N_n (the number of nodes) is determined by the data of interest. In this study, we used 84 nodes to correspond to the number of brain regions in the Desikan-Killiany atlas. In addition, 300 nodes were used in scale-free analysis to reduce the bias from finite network size.

The choice of ρ (controlling the node heterogeneity) has been explained in Methods. $\rho = 1$ was used in this study to maximize nodal heterogeneity while preserving the sequential arrangement of nodes along the perimeter.

N_a (the number of axons simulated) was fixed to $2e^5$. This value was chosen as a balance between the favor to large N_a and the computational affordability. The axon origins were sampled in a random manner; thus, a large N_a is favored to capture the connectivity distribution of generated networks. Consider two networks (connectivity matrices M_1 and M_2) that only differ in the number of axons simulated (N_a and cN_a, respectively; $N_a \to \infty$, c is a constant). The connectivity expectation would be $M_2 = cM_1$. This further guided the trick (linear scaling) used in parameter optimizations (See Parameter optimization section in supplementary materials).

The effects of R (circle radius), θ (angular constraint), and L_s (growth step length) on generated networks were closely related. Fig. S5 demonstrated a linear relationship between R and L_s. The association between θ and L_s is complex and non-linear; however, the ratio θ/L_s could be approximated as the angular changes that an axon can make per unit growth length. Therefore, we decided to fix R and θ and investigate the variations in L_s, making L_s a key tunable parameter in the model. R was set to a random constant ($R = 30$ was used), and the choice of $\theta = 15°$ was inspired by the angle values typically used between successive steps in tractography.

S_{max} (the maximum number of growing steps allowed) ensured that simulations could stop even if axons were trapped by the "black hole" zone. $S_{max} = 3R/L_s$ was used such that axons were able to connect furthest points on the circle (Euclidean distance of 2R), while a margin of R was included to allow curved axon trajectories.

β describes the distance decay of attractive force, controlling the relative contribution of guidance exerted by adjacent and distance nodes. As a result, values of β are closely related to topology of generated networks, and we make it a key tunable parameter of the model.

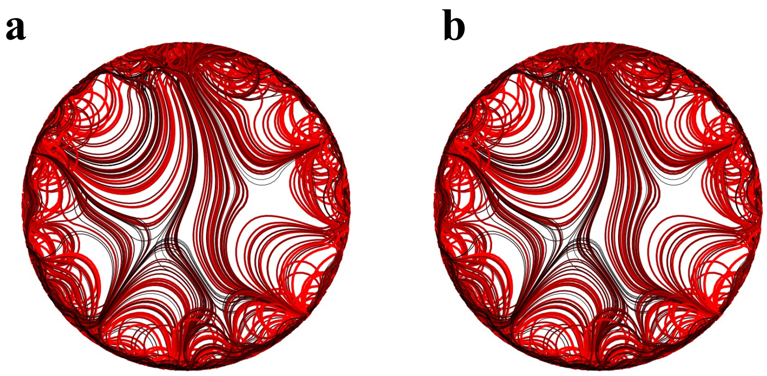

Figure S5. R and L_s are linearly related. *a) A network generated with R = 30 and $L_s = 1$. b) A network generated with R = 60 and $L_s = 2$. All other parameters, including the angular coordinates of nodes and axon origins, were the same. The two parameter combinations generated the same network.*

Stochasticity from node and axon coordinate sampling

Throughout the quantitative analyses of the manuscript, we considered ensembles of networks generated by random sampling of node coordinates. In Fig. 2a-d and 3a-d, we visualize the axon fiber structure, connectivity matrices, edge weight-distance association, and weight distributions for representative generated networks only. To facilitate visual comparison, all representative

networks in Fig. 2a-d and 3a-d were generated with the same set of node center / axon coordinates by fixing the random number generator in MATLAB. To demonstrate that these visualizations are robust to the inherent stochasticity in node/axon sampling, we show the results from a different set of node center / axon coordinates in Fig. S6 and S7.

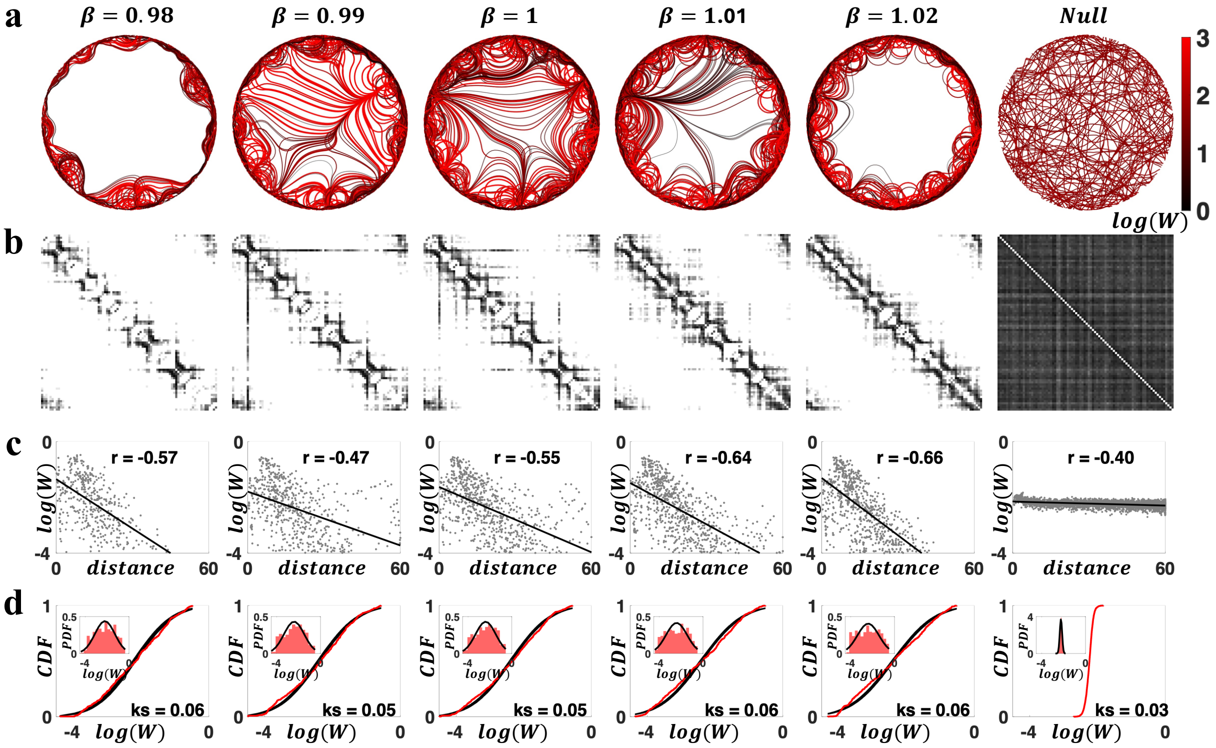

Figure S6. Same as Fig.2 but generated with a different set of random number generator.

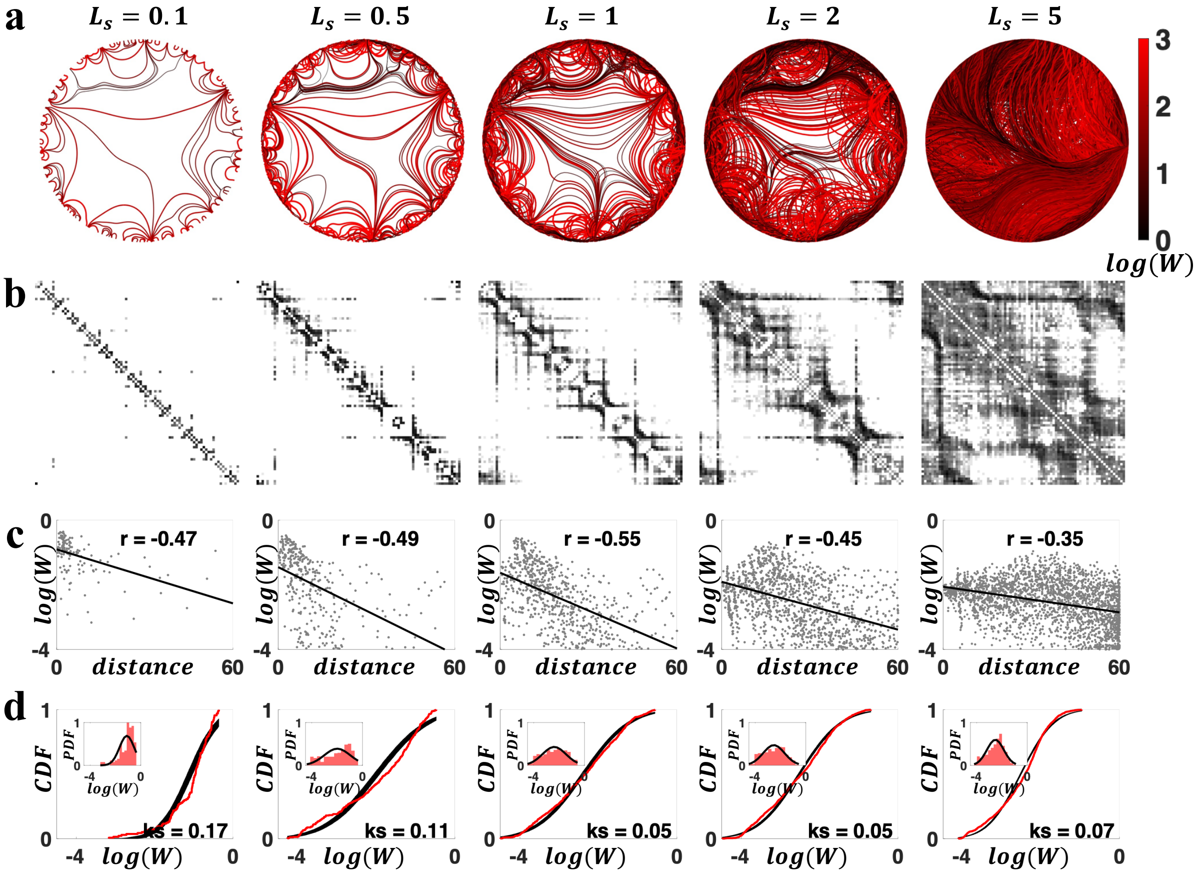

Figure S7. Same as Fig.3 but generated with a different set of random number generator.

Variance in fiber lengths explained by Euclidean distances

Previous work found that the variance in fiber lengths explained by Euclidean distances is inconsistent between studies, ranging from 22-79%. Despite the wide range reported, the results from our model generated axons fell within the range (generally between 70-80%), whereas values from the random walk null model generated axons were much larger (Fig. S8).

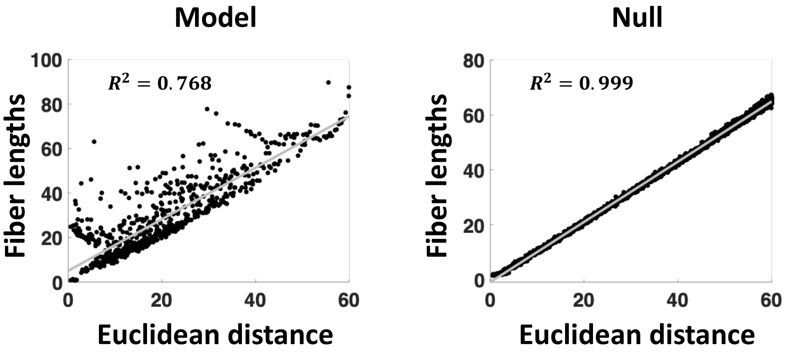

Figure S8. Variance in fiber lengths explained by Euclidean distances is consistent with empirical observations in model but not in null.

Weight distributions (null model, and alternative distributions ks test results)

In the main text, using representative parameter combinations, we suggested that the model was able to simulate networks whose connection weights were best described as lognormal distributions. Here, we statistically compared the fitted KS statistics among candidate distributions (lognormal, normal, gamma, exponential, Weibull, Fig. S9). 50 networks were generated for each parameter combination, and the KS statistic of fit were compared between distributions using paired t-tests. Lognormal distribution was found to be the best fit (significantly smaller KS statistic compared to other distributions, all $p < 0.05$) in networks generated with $\beta = 0.98, 0.99, 1, 1.01,$ and 1.02 (L_s was fixed to 1), and $L_s = 1$ and 2 (β was fixed to 1). It was also the best fit in networks generated with the group average parameter for the HCP population, despite the KS statistic difference between lognormal and Weibull distributions were not significant.

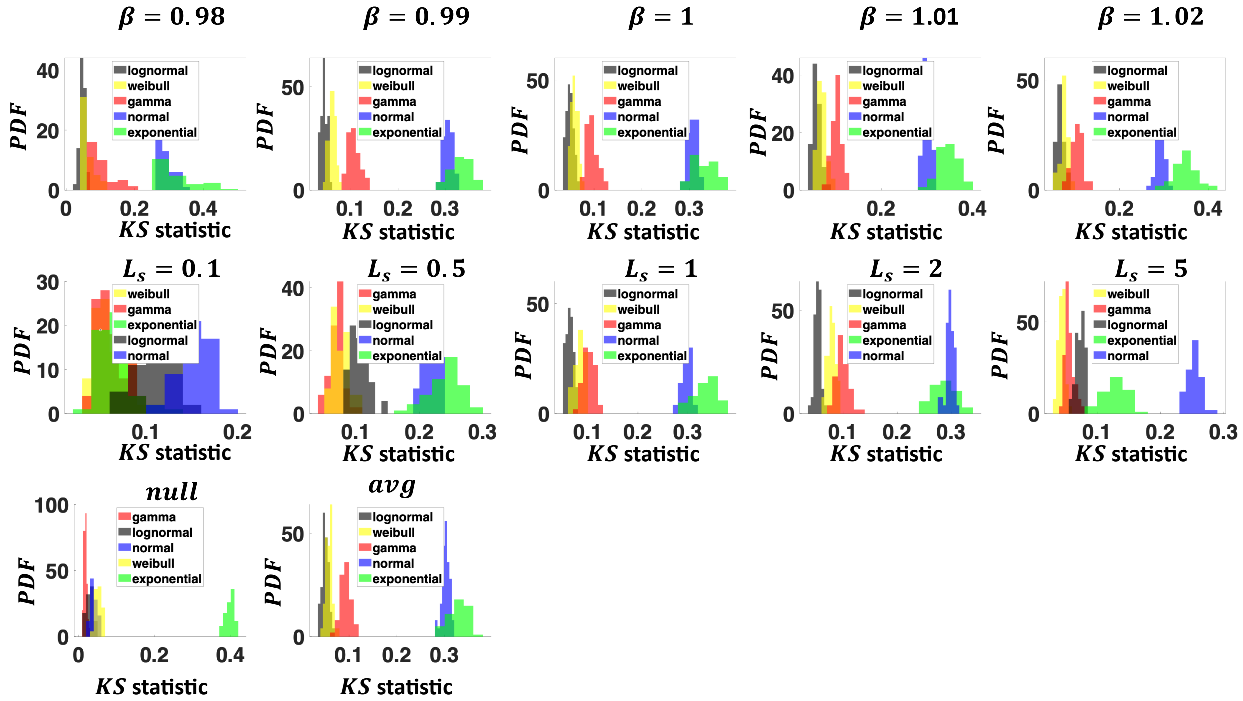

*Figure S9. **KS statistics of fitted candidate distributions.** Legends were ranked by ascending mean KS statics.*

It should be noted that connection weights in null networks were not best described as a log-normal distribution, despite the KS statistic is small (Fig.2 and Fig. S9). To better characterize the difference between model and null networks, distributions of normalized weights were shown in Fig. S10. Connection weights in null networks exhibited less variability (strong connections are rare) relative to in model networks.

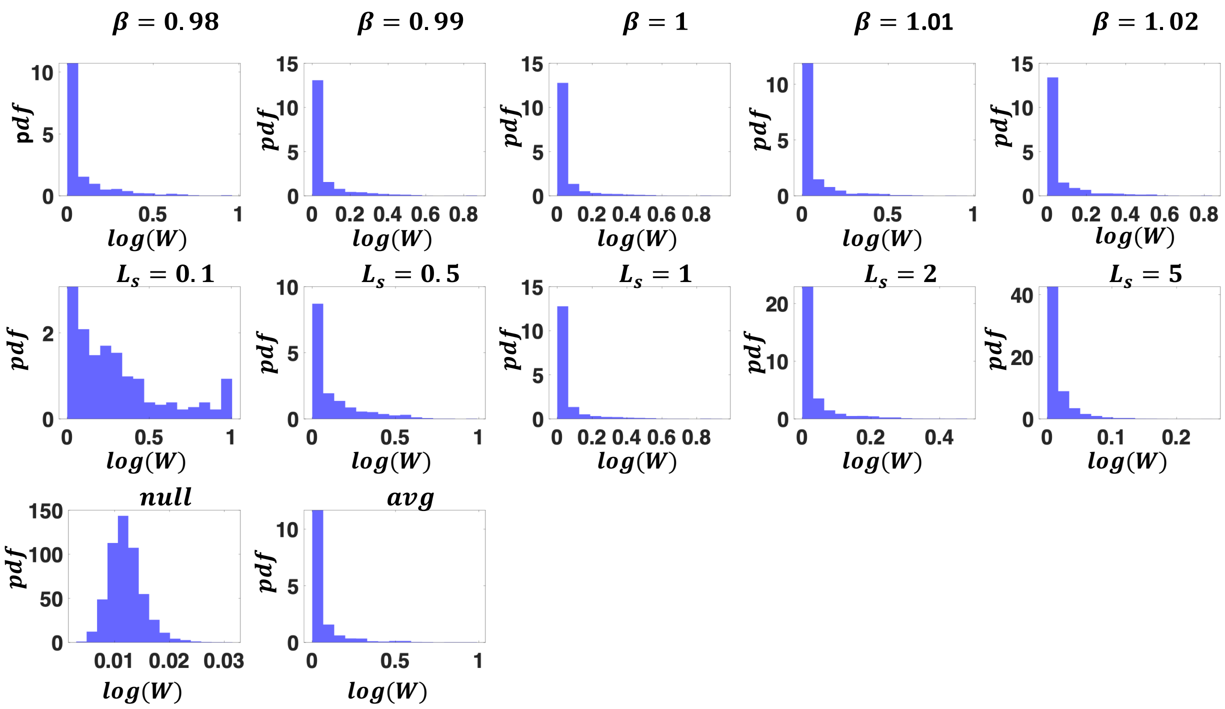

Figure S10. Probability density function of weight distributions (normalized by nodal strengths) in simulated networks. Weights in null networks were small, denoting a lack of strong connections. In contrast, strong weights could be found in model networks.

p-value distributions of scale-free test

In the main text, we suggested that the model was able to generate networks with scale-free degree distribution. The conclusion was drawn from the observations that more than 50% of networks generated by a parameter combination showed a $p > 0.1$ in the scale-free test. Fig. S11 showed a histogram of p-values in 1,000 generated networks, for each evaluated parameter combination. Null networks did not show a scale-free property. Scale-free was evident in $\beta =$ 0.98 and 1 (L_s was fixed to 1), and all L_s values considered (β was fixed to 1). The group

average parameters of HCP population were found to be able to generate scale-free networks, despite the p value marginally above the threshold of $p = 0.1$.

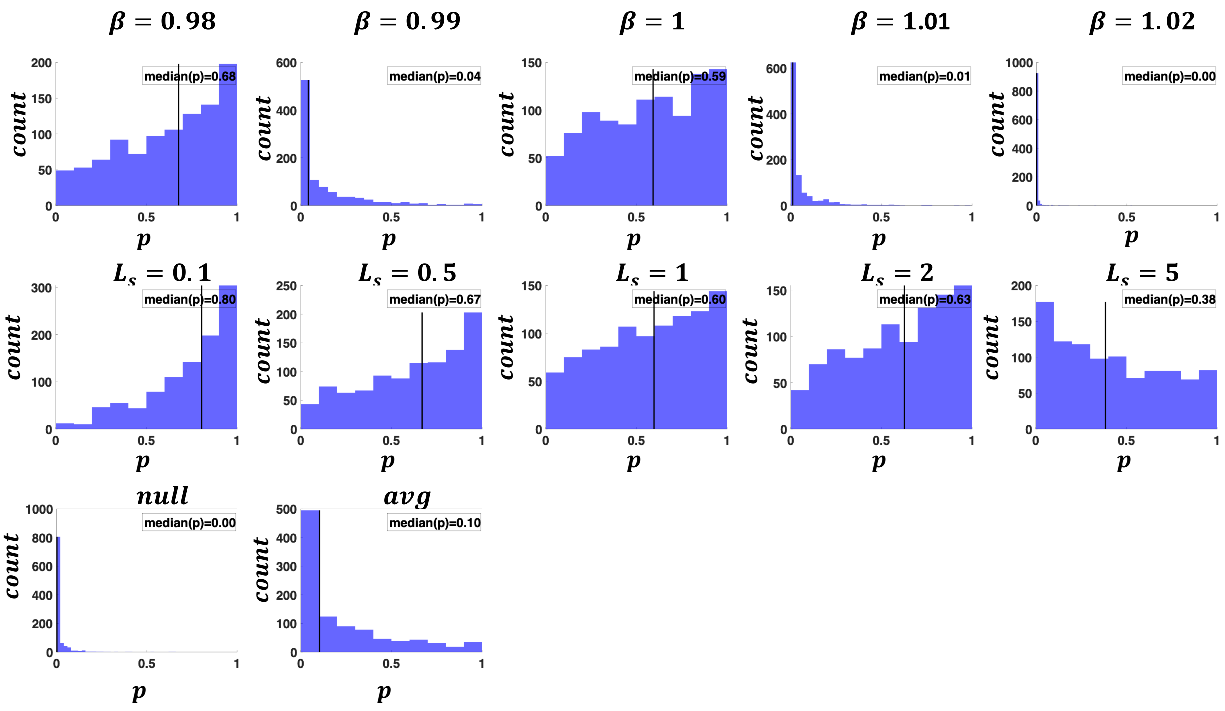

*Figure S11. **p value distributions of scale-free tests.** Each histogram displayed p values of 1,000 networks.*

The effect of network density on weight measures

In Figs. 2 and 3 we evaluate the weight properties of generated networks for a range of model parameters. These generated networks are not thresholded and span a variety of network densities. A related concern is whether the variations in generated networks are driven by inconsistent network densities, given network density can fundamentally impact other network properties. To answer the question, we threshold networks evaluated in Figs. 2 and 3 to a

network density of 10% and visualize the weight-distance association as well as weight distributions in Fig. S12, S13. In brief, observations remain similar after thresholding, with the exception of $L_s = 5$ (Fig. S13a). However, this parameter setting does not generate brain-like networks. We conclude that variations in network density are intrinsic to the generative process and are not due to the discarding of self-connections and unsuccessful axons.

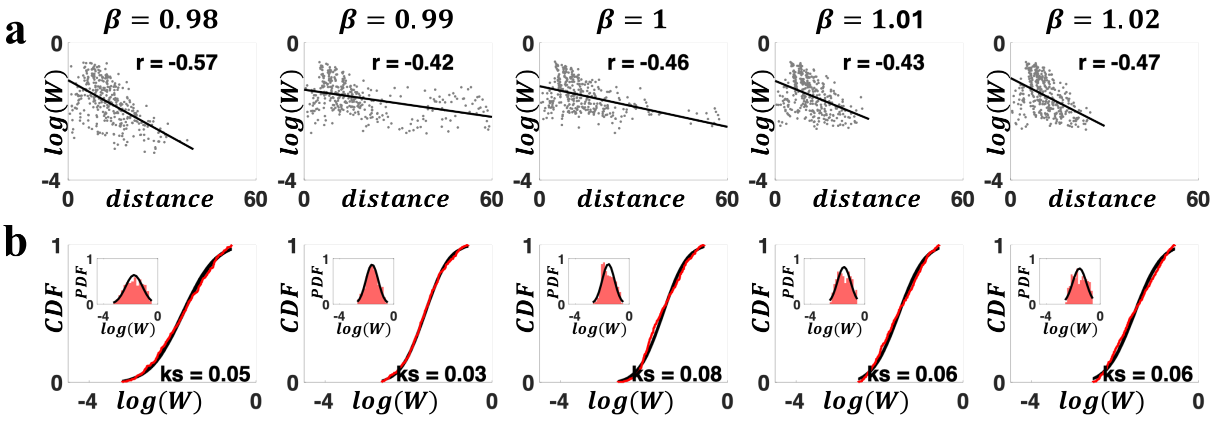

Figure S12. Edge weight measures in thresholded networks generated with a variety of β. a) Associations between edge weights and distances. b) Edge weight distributions. All networks are threshold to a density of 10%.

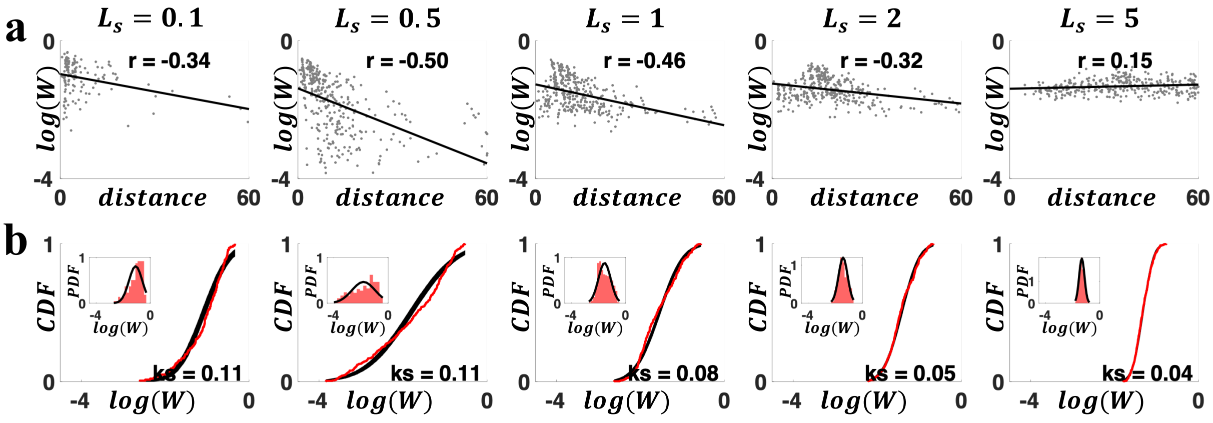

Figure S13. Edge weight measures in thresholded networks generated with a variety of L_s. a) Associations between edge weights and distances. b) Edge weight distributions. All networks are threshold to a density of 10%.

Contour plot patterns of topological properties are insensitive to network density

In Fig. 4, we evaluated the weighted topological properties of generated networks at a network density of 10%. We found that the patterns of contour plots are insensitive to network density and show the results for network density of 5% in Fig. S14.

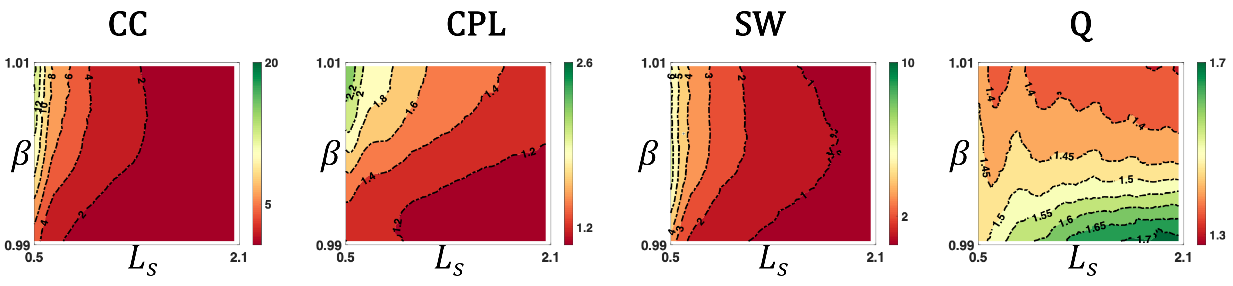

Figure S14. Weighted topological properties (the same metrics as in Fig. 4a) evaluated at 5% network density.

Raw values of topological metrics

In Fig. 4a, we showed the weighted topological properties of generated networks, normalized by weight and degree preserved null networks. Here in Fig. S15, we show the raw values of weighted topological properties of CC, CPL, and Q. SW is not included here because it is by definition normalized. It should be noted that CC and modularity Q changed with parameters in a different manner compared to Fig.4.

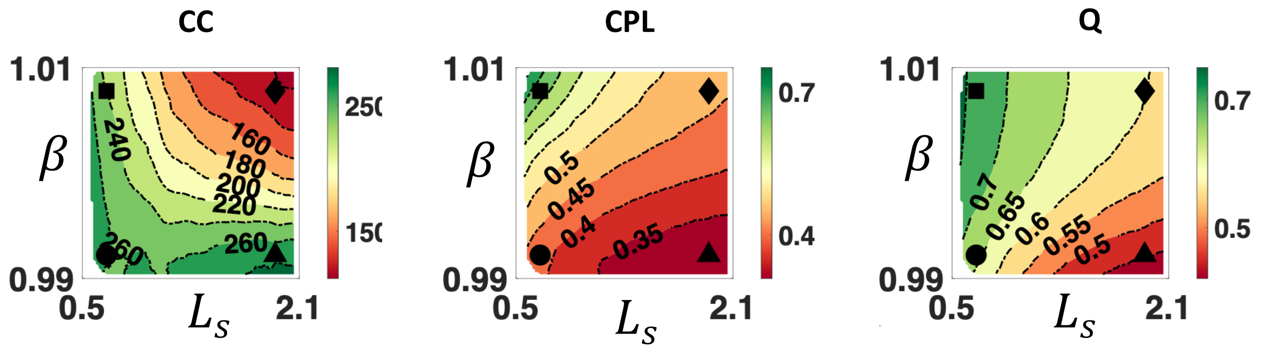

Figure S15. Raw values of weighted topological properties (CC, CPL, and modularity Q).

Node modular assignment in generated networks

In Fig. 4 we visualized the modularity Q and connectivity matrices for example generated networks. Fig. S16 visualizes the modular assignment of nodes.

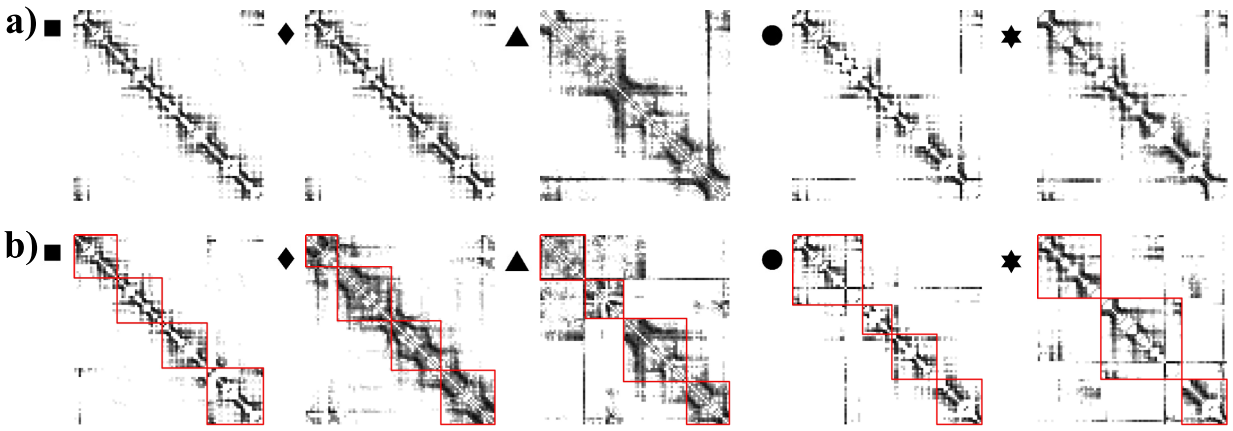

Figure S16. Modular assignment of generated networks in Fig. 4. a) Same as Fig. 4c where nodes are ordered according to spatial embeddings. b) Connectivity matrices rearranged for module visualization. Modular blocks are labelled by red lines.

Modified energy cost relative to the state-of-the-art models

In this work, we developed a new parameter fitting cost function to fit the model parameters to individual connectomes. This is because the energy cost used in the current state-of-the-art models is not applicable to our model, due to the missing nodal correspondence between generated and empirical connectomes. However, three topological properties considered in the energy function, including the degree, clustering, and betweenness centrality, are still eligible for comparison. Thus, we modified the energy cost function by considering these three features only and compared the energy achieved by our model, compared to the state-of-the-art matching index model and pure geometric model.

As shown in Fig. S17, our model achieved a better fit compared to the geometric model, and a worse fit relative to the matching index model. This result is expected: The geometric model has the lowest model complexity (with only one model parameter). Although the matching index and our model both have 2 model parameters, our model relies on purely geometric information, where the matching index model used both geometric and topological information. In conclusion, geometry-dependent dynamic axon guidance generates networks better replicate empirical connectome than a simple distance rule, whereas this improvement is smaller than considering complex topological information.

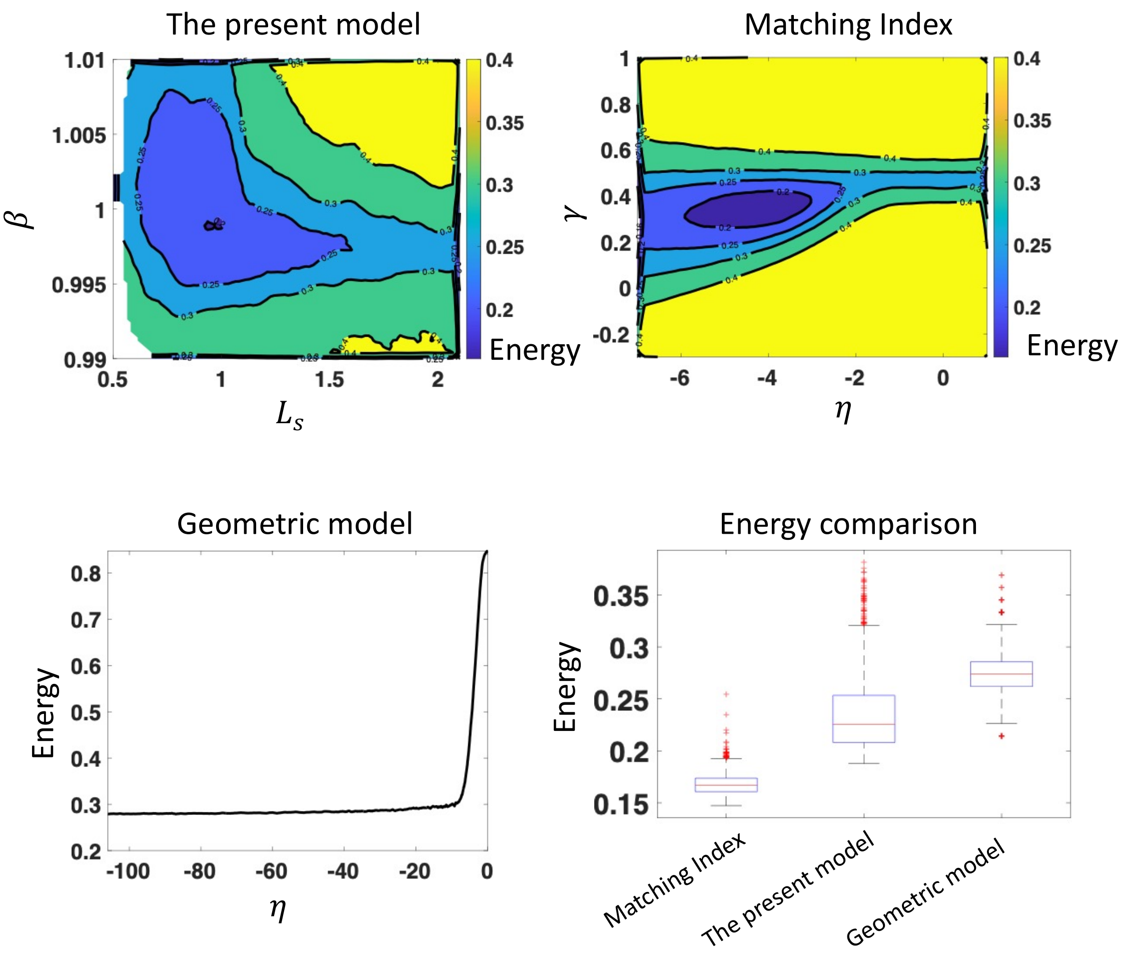

Figure S17. Our model achieved a better energy compared to the state-of-the-art geometric model, and a worse energy compared to the state-of-the-art matching index model. All energy functions consider degree, clustering, and betweenness centrality only.

Parameter optimization: linear scaling and model fit

As discussed above (the section explained the parameter specification of N_a), because axon sampling is probabilistic, the expectation of an edge weight should linearly scale with N_a. Thus, to optimize the model parameters, we linearly scaled the total connectivity of all networks (both model and empirical networks) to the same value.

A related concern is that this may disrupt the inter-individual variations in total connectivity. To test this, we randomly selected a sample of 100 participants from the HCP cohort. Model networks were linearly scaled to the total connectivity matching each individual before parameter optimization. We found that fitted parameters derived from the two approaches were comparable.

As per previous studies, we report the cost value of selected model networks in Fig. S18. Selected networks showed an RMSE of around 0.5 standard deviations on average, with comparable values among clustering coefficient, CPL, and modularity.

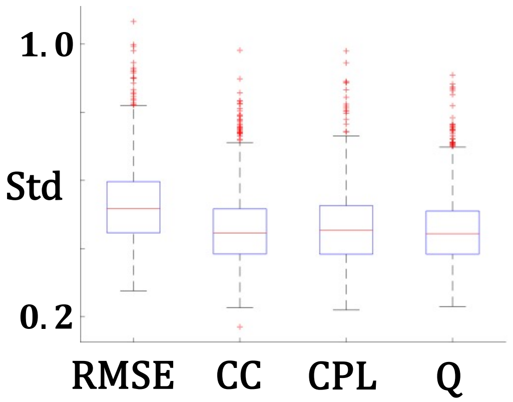

Figure S18. RMSE, and cost in CC, CPL, and modularity Q, of networks selected to optimize parameters for the HCP cohort.

Associate model parameters to individual traits

We optimized the model parameters for individual connectomes participating in the HCP dataset and associate them (β, L_s) to age and sex. No significant association was found to age, probably because HCP is a healthy young adult dataset in which effects of development and aging are less

evident. Males and females significant differ in L_s (Fig. S19), suggesting model parameters can capture inter-individual variations in connectomes. Future work could investigate the age-parameter association in aging and/or developing cohorts.

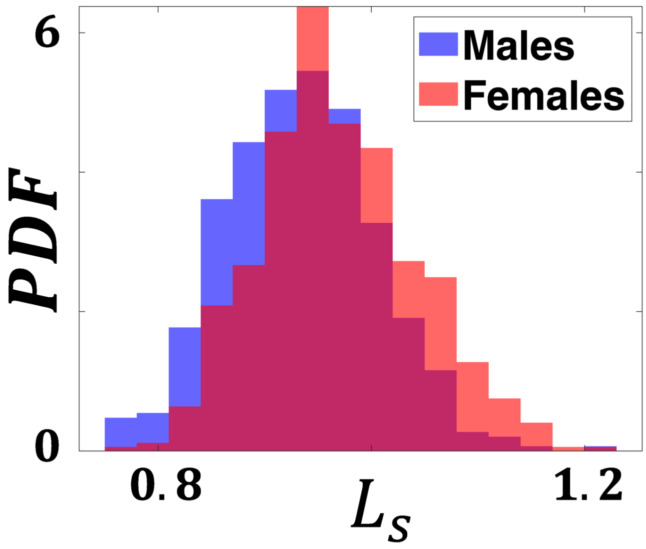

Figure S19. Males and females significantly differ in L_s (p<0.05, cohen's d = 0.49).
